# Primary Post-Traumatic *Aspergillus fumigatus* Soft-Tissue Infection of the Hand Requiring Digital Amputation and Reconstruction: A Case Report

**DOI:** 10.3390/jcm15218667

**Published:** 2026-11-06

**Authors:** Anna-Maria Mitropoulou, Francesk Mulita, Panagiotis Dimitrios Papadopoulos, Dimitrios Litsas, Vasileios Leivaditis, Panagiotis Antzoulas, Antonios Katsantonis, Helen Bolanaki, Anastasios J. Karayiannakis

**Affiliations:** 1 Second Department of Surgery, Medical School, Democritus University of Thrace, 68100 Alexandroupolis, Greeceant.katsas@gmail.com (A.K.);; 2 Department of General Surgery, Spital Herisau, 9100 Herisau, Switzerland; 3 Department of Surgery, General Hospital of Lamia, 35100 Lamia, Greece; dimlitsas@icloud.com; 4 Department of Cardiothoracic and Vascular Surgery, Westpfalz Klinikum, 67655 Kaiserslautern, Germany; 5 Department of Orthopaedics, University Hospital of Patras, 26504 Patras, Greece

**Keywords:** *Aspergillus fumigatus*, hand infection, traumatic inoculation, soft-tissue infection, diabetes mellitus, surgical debridement, digital amputation, reconstruction

## Abstract

Background: Primary post-traumatic *aspergillosis* is an uncommon fungal infection resulting from direct inoculation of Aspergillus species into disrupted skin and soft tissue. Although it is more frequently encountered in immunocompromised patients, severe localized infection may also occur after traumatic inoculation, particularly in the presence of previously unrecognized predisposing conditions. Involvement of the hand is rare. Case Presentation: A 68-year-old man was referred to our department with a progressive necrotizing infection of the right hand following an occupational agricultural injury. The initial injury had been surgically treated at another institution. On presentation, extensive necrosis involved the palmar aspect of the hand; the index, middle, and ring fingers; and the hypothenar region. The patient had no known systemic condition predisposing him to fungal infection at presentation. During hospitalization, however, persistent hyperglycemia led to the diagnosis of previously unrecognized and uncontrolled diabetes mellitus. Surgical exploration revealed extensive soft-tissue necrosis, thrombosed vessels, poorly perfused and friable tendinous structures, and exposed bony structures of the middle and ring fingers. *Aspergillus fumigatus* was repeatedly isolated from wound and deep-tissue specimens. Antifungal therapy was initially started with amphotericin B but was discontinued because of a severe allergic reaction and replaced with voriconazole. Three sequential surgical debridements were performed. Magnetic resonance imaging demonstrated extensive soft-tissue involvement without evidence of osteomyelitis. A fourth operation combined further debridement with amputation of the irreversibly damaged middle and ring fingers and immediate reconstruction of the resulting defect with local flap coverage. Subsequent cultures were negative, and the postoperative course was uncomplicated. Conclusions: Post-traumatic *A. fumigatus* infection of the hand is rare but may lead to extensive soft-tissue destruction. An invasive fungal infection should be considered in progressive necrotizing wounds following contaminated trauma, particularly when the clinical course is atypical or refractory to conventional antibacterial treatment. Early microbiological diagnosis, systemic antifungal therapy, repeated surgical debridement, and recognition of underlying predisposing conditions are essential for successful management.

## 1. Introduction

Aspergillus species are ubiquitous filamentous fungi that can cause a wide spectrum of disease, ranging from localized cutaneous infection to invasive and disseminated *aspergillosis*. *Primary cutaneous aspergillosis* (PCA) develops when Aspergillus conidia are directly inoculated into disrupted skin or soft tissue. In contrast, secondary cutaneous *aspergillosis* results from hematogenous dissemination or direct extension from an infection at another site [1,2,3].

Direct inoculation has been associated with penetrating trauma, burns, surgical wounds, intravenous catheter sites, and exposure of damaged tissue to contaminated environmental material [1,2,3]. Although PCA is most often described in immunocompromised patients, localized infection may also occur in individuals without a previously recognized immunodeficiency, particularly following trauma or surgery [1,2,3].

Post-traumatic *aspergillosis* of the hand is particularly uncommon. Previous reports of traumatic or localized hand infection have involved species such as *A. flavus* and *A. terreus* [4,5,6]. Post-traumatic infections caused by *A. fumigatus* have also been reported, but mainly in different anatomical or clinical settings, including traumatic wound and fracture-related infections [7,8].

We report a case of primary post-traumatic *A. fumigatus* soft-tissue infection of the hand with extensive necrotizing destruction involving multiple digits. The clinical course was further complicated by the diagnosis of previously unrecognized uncontrolled diabetes mellitus and ultimately required repeated surgical debridement, digital amputation, and simultaneous reconstruction.

## 2. Case Presentation

A 68-year-old man was referred to our Plastic Surgery Department with a progressive necrotizing infection of the right hand following an occupational agricultural injury.

The initial injury had been surgically managed at another institution, where the resulting soft-tissue defects were treated with partial- and full-thickness skin grafts. Despite initial treatment, progressive soft-tissue necrosis developed, prompting referral to our department.

According to the available referral history, the hand had become caught in a belt mechanism of agricultural machinery. Primary closure had initially been attempted where possible, followed by split- and full-thickness skin grafting. Necrosis subsequently affected the grafted areas of the palm, particularly near the middle, ring, and little fingers. The patient was referred to our department in early September; the exact date of the injury and the interval before referral could not be established from the available history.

**Figure 1:**
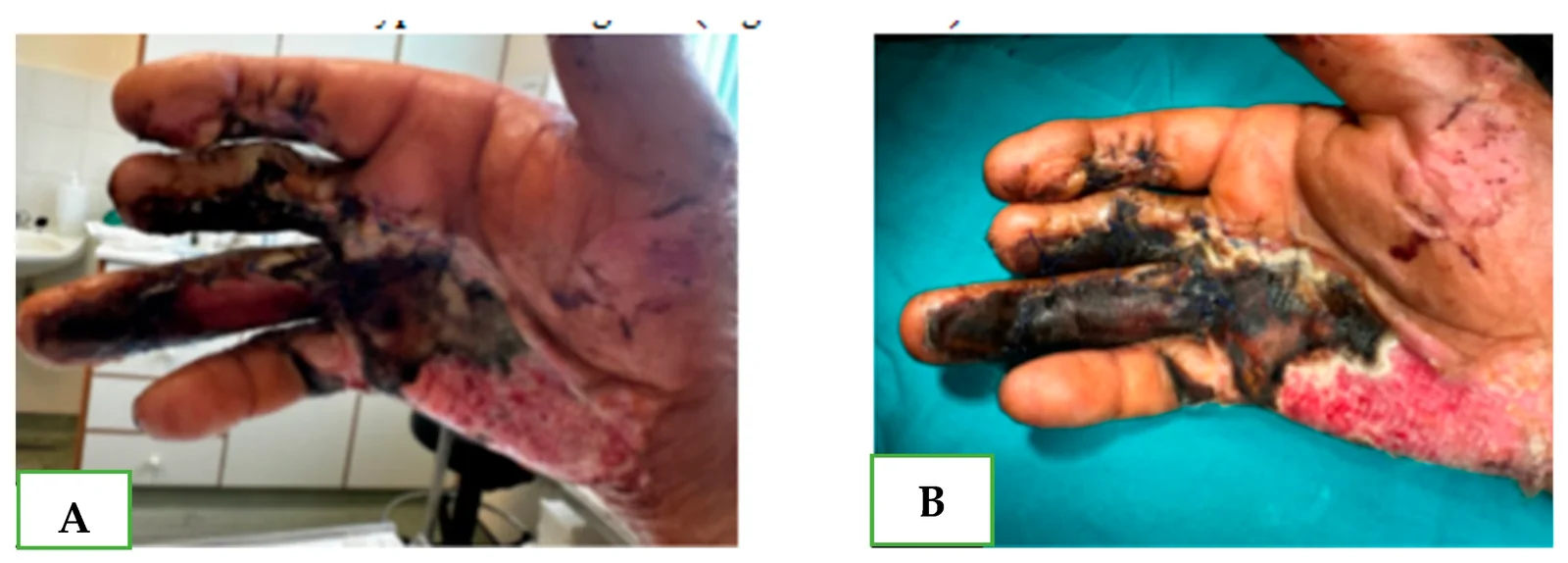
Initial clinical presentation. (**A**) Palmar view of the right hand at presentation to our department, demonstrating extensive necrotic changes involving the palm and multiple digits. (**B**) Preoperative appearance before the first surgical debridement, showing extensive areas of soft-tissue necrosis.

On examination, extensive necrotic changes involved the palmar aspect of the right hand, including the thumb, index, middle, and ring fingers and fifth digit, with additional involvement of the hypothenar region (Figure 1A,B).

At presentation, the patient had no known systemic condition predisposing him to fungal infection. During hospitalization, however, persistent hyperglycemia was noted on routine laboratory testing, leading to the diagnosis of previously unrecognized and uncontrolled diabetes mellitus.

Daily blood glucose measurements were repeatedly above 150 mg/dL, and HbA1c was 10.2%. Endocrinology consultation was obtained, and glucose-lowering treatment was adjusted during admission, with subsequent improvement in glycemic control.

**Figure 2:**
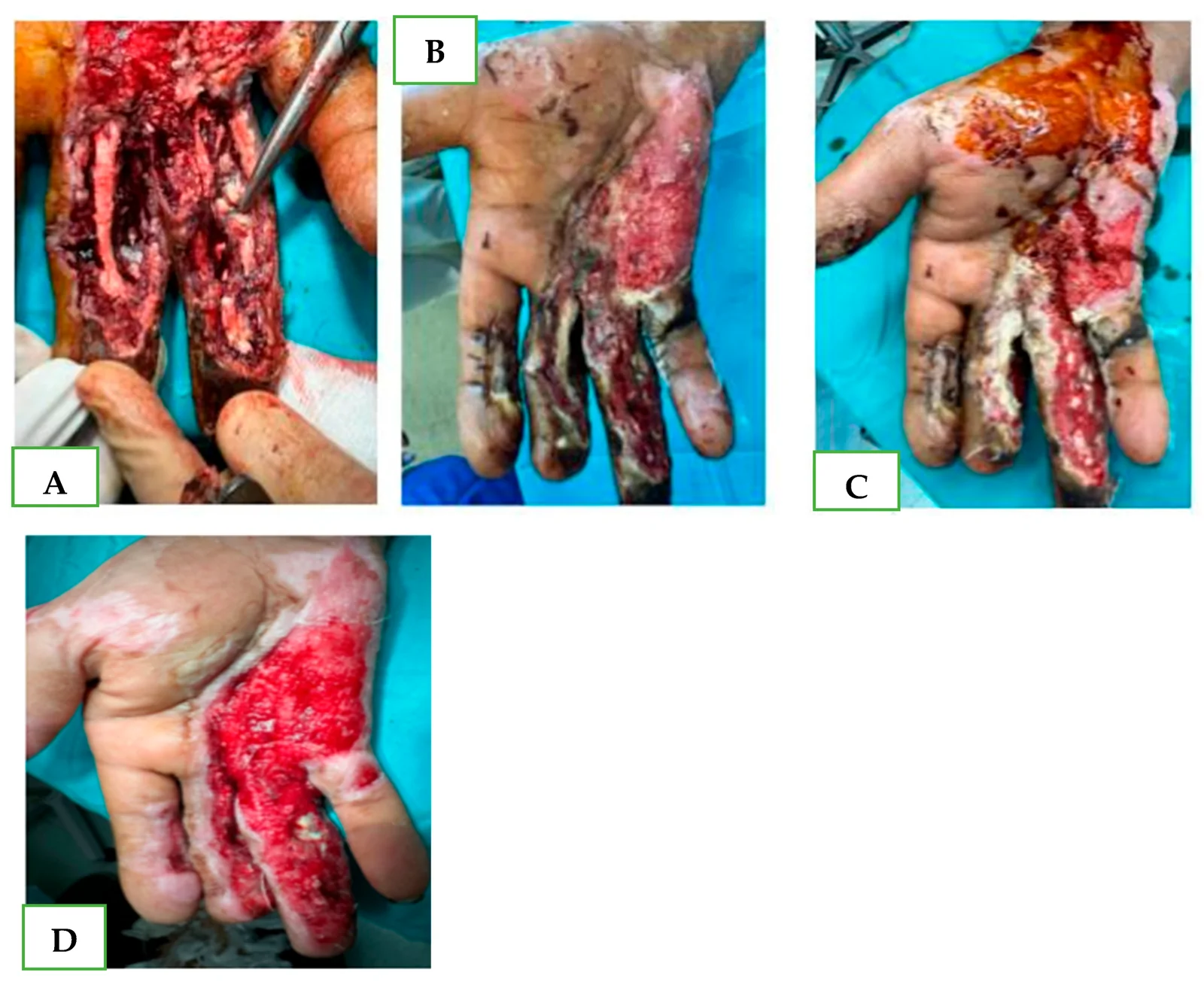
Serial surgical debridement of the right hand. Representative clinical and intraoperative photographs demonstrating the progression of tissue necrosis and sequential excision of devitalized tissue during the first, second, and third surgical debridements. Intraoperative views demonstrate extensive involvement of the deep soft tissues and tendinous structures. (**A**) First debridement. (**B**) Before 2nd debridement. (**C**) After 2nd debridement. (**D**) Before/at 3rd debridement.

Because of the progressive necrosis, surgical exploration and extensive debridement were undertaken. Intraoperative findings included widespread necrotic eschar and devitalized soft tissue. Several vessels were thrombosed, and the involved tendinous structures were friable and poorly perfused. Tissue destruction was particularly severe in the middle and ring fingers, with exposure of the underlying bony structures (Figure 2A).

Microbiological specimens, including wound swabs and deep-tissue samples, were obtained during the surgical procedures. *Aspergillus fumigatus* was repeatedly isolated from these specimens, supporting the diagnosis of a clinically significant post-traumatic fungal infection.

In addition to samples taken at surgery, wound swabs were collected before and after local wound care and dressing changes during the early course of treatment, including the interval between the first two debridements. Histopathological examination of the excised tissue was not performed.

Broad-spectrum antibacterial therapy with piperacillin–tazobactam and daptomycin was administered concomitantly. Systemic antifungal therapy was initially started with amphotericin B. Because of a severe allergic reaction, amphotericin B was discontinued and replaced with voriconazole.

Before the fungal culture results became available, empirical antibacterial treatment had included cefoxitin (Mefoxil) and metronidazole (Flagyl). Voriconazole was administered intravenously for at least four weeks under Infectious Diseases supervision; the precise administered dose and total treatment duration could not be verified from the available information.

Persistent areas of devitalized tissue necessitated three sequential surgical debridements, with progressive removal of nonviable (Figure 2A–D). At each procedure, nonviable tissue was removed and further specimens were obtained for microbiological examination.

The three initial debridements were followed by a fourth procedure for further excision, digital amputation, and reconstruction. The treatment course extended from early September to late October; exact dates and intervals between operations were not available.

Following the third debridement, magnetic resonance imaging demonstrated extensive soft-tissue damage without evidence of osseous involvement. Despite the absence of osteomyelitis, the degree of soft-tissue destruction affecting the middle and ring fingers was considered irreversible.

**Figure 3:**
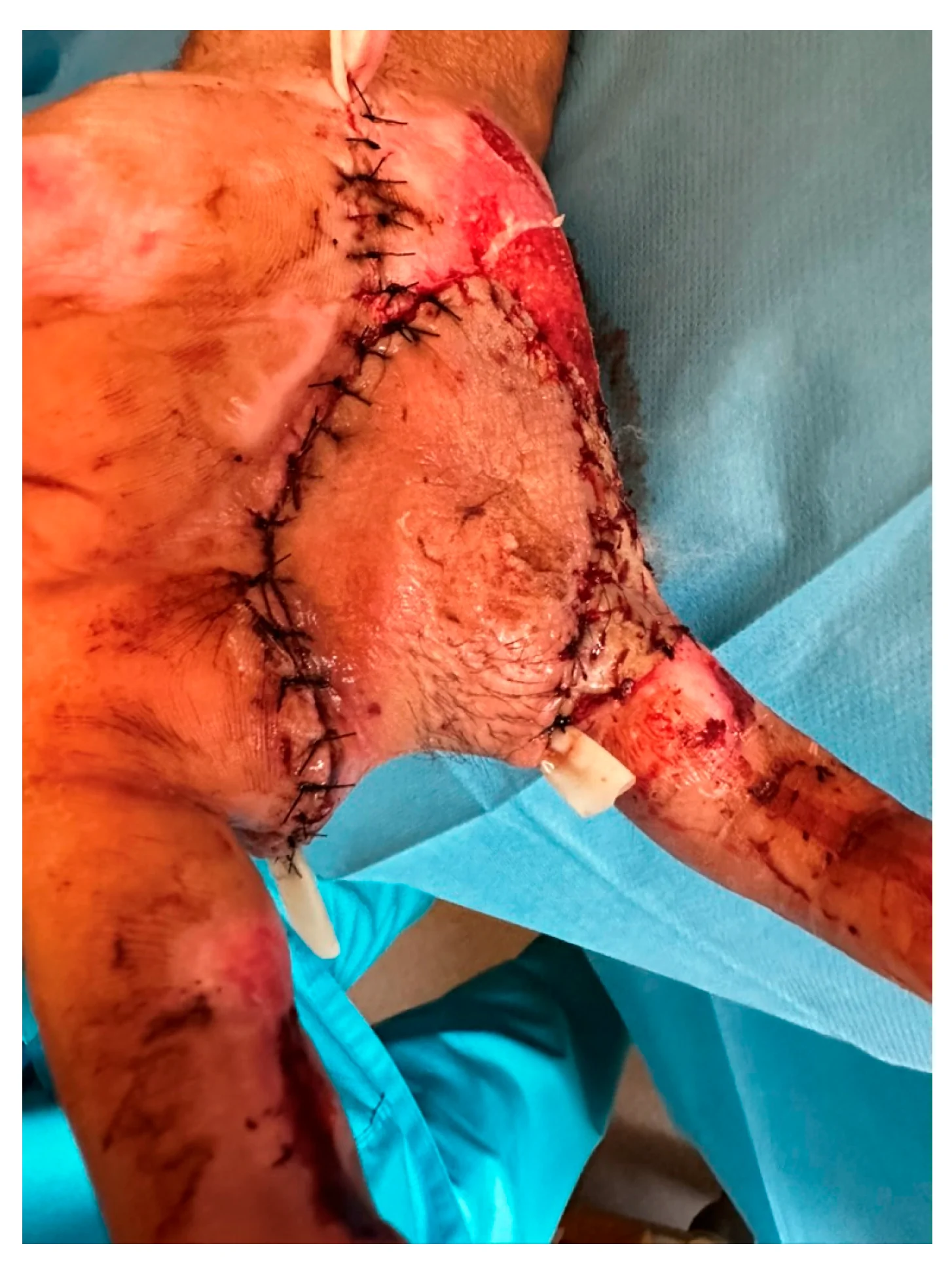
Definitive surgical management and reconstruction. Appearance of the right hand following further surgical debridement and amputation of the irreversibly damaged middle and ring fingers. The clinically viable dorsal skin of the ring finger was preserved during amputation and used to provide coverage of the resulting palmar soft-tissue defect.

A fourth operation was therefore performed. During the same procedure, residual nonviable tissue was further debrided, and the irreversibly damaged middle and ring fingers were amputated. As the dorsal skin of the ring finger remained clinically viable, it was preserved during amputation and used to provide coverage of the resulting palmar soft-tissue defect (Figure 3).

The remaining bony segments of the middle and ring fingers had become exposed and detached from the surrounding viable soft tissues after serial debridement. Their removal was therefore considered necessary, while the viable dorsal skin of the middle finger was rotated to cover the palmar defect.

**Figure 4:**
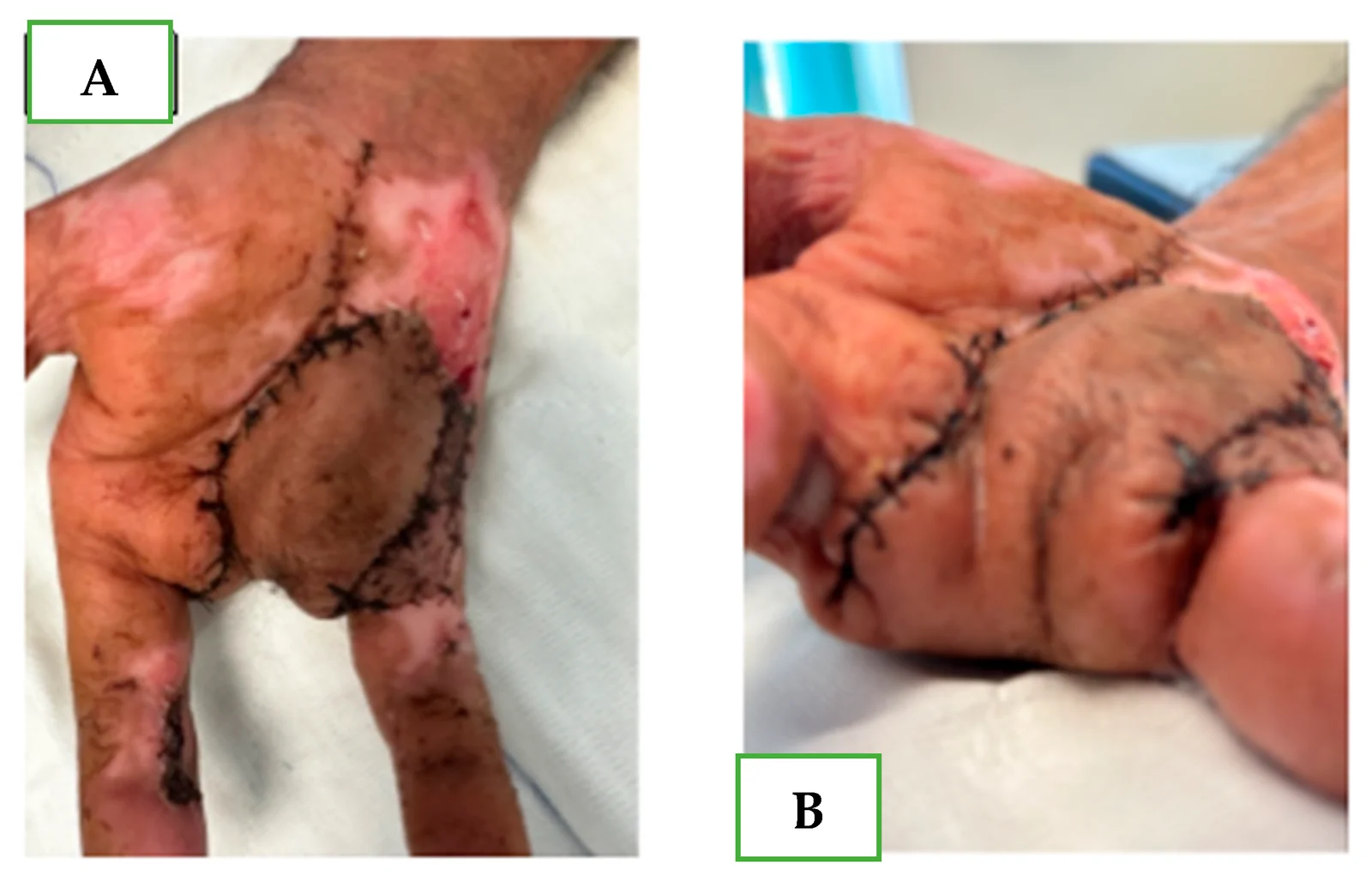
Early postoperative outcome. (**A**,**B**) Palmar and oblique views of the reconstructed right hand during postoperative wound assessment before suture removal, demonstrating viable flap coverage and ongoing wound healing.

Subsequent microbiological cultures were negative for fungal growth. The postoperative course was uncomplicated, and the patient was discharged in good general condition following postoperative monitoring. He was subsequently followed in the outpatient clinic with wound assessments and dressing changes every 2 days. Satisfactory wound healing and viability of the reconstructed soft-tissue coverage were observed, with no clinical evidence of recurrent local infection. Sutures were removed on postoperative day 14 (Figure 4A,B).

Postoperative monitoring and physiotherapy were undertaken during the first seven days. The patient returned for a further examination ten days after suture removal, with no clinical complications identified at that visit. Longer-term functional assessment and recurrence data were not available.

## 3. Discussion

Primary *aspergillosis* of the skin and soft tissues is an uncommon manifestation of Aspergillus infection and usually results from direct inoculation of fungal conidia through a disrupted cutaneous barrier [1,2,3]. Trauma provides a potential route of entry, particularly when an open wound is exposed to soil, vegetation, dust, or other contaminated environmental material.

The rarity of PCA was highlighted by Tatara et al., who reviewed 130 cases reported between 1967 and 2015 [1]. Their analysis demonstrated considerable variation in host characteristics, causative Aspergillus species, treatment, and outcome, while emphasizing the importance of underlying systemic conditions in the clinical course of the disease.

Host factors were particularly relevant in our patient. At presentation, there was no known history of diabetes mellitus or another major systemic condition predisposing him to fungal infection. Persistent hyperglycemia during hospitalization, however, led to the diagnosis of previously unrecognized uncontrolled diabetes mellitus. Accordingly, describing the patient as simply “immunocompetent” would be misleading. Diabetes may adversely affect host defense, microvascular function, and wound healing and may therefore have contributed to the severity and persistence of the infection.

The HbA1c value of 10.2% indicated substantial pre-existing dysglycemia, although its contribution to the severity of the local infection cannot be determined from this single case.

Only a small number of Aspergillus infections involving the hand have been reported. Epstein et al. described primary cutaneous *A. flavus* infection of the hand in a patient with non-insulin-dependent diabetes mellitus who was successfully treated with oral itraconazole [4]. Türkşen et al. reported post-traumatic infection involving the middle finger in a 70-year-old man after a woodcutting injury; histopathology demonstrated fungal elements, and cultures yielded *A. flavus* together with *Candida guilliermondii* [5].

More recently, Ing et al. described primary traumatic *aspergillosis* of the hand caused by *A. terreus* following a mould-contaminated penetrating injury [6]. That patient also had poorly controlled diabetes mellitus and improved following surgical debridement, voriconazole therapy, and optimization of glycemic control. These reports support traumatic inoculation as a mechanism of primary Aspergillus infection of the hand, although the causative species differed from that identified in our patient.

Post-traumatic infection caused specifically by *A. fumigatus* has been described in other clinical settings. Gettleman et al. reported an invasive *A. fumigatus* wound infection following trauma [7]. Mertens et al. subsequently described *A. fumigatus* fracture-related infection following a severe open tibial fracture and reviewed previously reported cases of Aspergillus osteomyelitis [8,9]. In contrast to these fracture-related infections, magnetic resonance imaging in our patient showed extensive soft-tissue destruction without evidence of osseous involvement.

Our case is therefore notable for the combination of direct post-traumatic infection of the hand, repeated isolation of *A. fumigatus*, extensive necrotizing destruction involving multiple digits, previously unrecognized uncontrolled diabetes mellitus, and the need for repeated debridement followed by digital amputation and immediate reconstruction. Although post-traumatic *A. fumigatus* infection has been reported previously, this combination of anatomical location, causative organism, severity of soft-tissue destruction, and reconstructive requirements appears to be exceptionally uncommon.

Diagnosis can be challenging because the early manifestations of cutaneous and soft-tissue *aspergillosis* are nonspecific. Progressive necrosis in a traumatic wound may initially be attributed to bacterial infection or tissue ischemia. Failure to improve despite appropriate antibacterial treatment should therefore prompt consideration of an invasive fungal infection, particularly after environmental contamination.

Adequate microbiological sampling is essential. In our patient, *A. fumigatus* was repeatedly isolated from wound and deep-tissue specimens in the setting of progressive tissue necrosis, supporting its clinical significance rather than incidental colonization.

An important diagnostic limitation is the absence of histopathological examination to demonstrate fungal invasion of tissue. Although repeated positive deep-tissue cultures in the setting of progressive necrosis favored a clinically significant infection, culture findings alone cannot conclusively distinguish invasion from colonization or exclude other filamentous fungi.

Management depends on the extent of infection, the causative species, host factors, and response to treatment. Published reports support a combination of systemic antifungal therapy and surgical excision of infected or devitalized tissue in extensive disease [1,3,6]. In the present case, amphotericin B had to be discontinued because of a severe allergic reaction, and treatment was continued with voriconazole.

The patient underwent Infectious Diseases assessment and thoracic CT imaging, together with serial chest radiographs approximately every four to five days. The imaging findings and any serum fungal biomarker results were not available for inclusion in this report.

Antifungal therapy alone, however, could not address the extensive burden of necrotic and poorly perfused tissue. Three sequential debridements were required, followed by a fourth operation combining further excision of residual nonviable tissue with amputation of the irreversibly damaged middle and ring fingers and immediate reconstruction of the resulting defect.

From a reconstructive perspective, this case illustrates the difficult balance between adequate source control and preservation of hand function. Irreversibly devitalized structures must be removed to control infection, while unnecessary loss of viable tissue should be avoided. In our patient, simultaneous reconstruction during the fourth procedure provided stable soft-tissue coverage after further debridement and digital amputation. Subsequent negative cultures and satisfactory wound healing supported successful local control of the infection.

Amputation was selected because of the extensive loss of viable tendinous and surrounding soft-tissue support and the exposure of detached skeletal segments, rather than MRI-confirmed osteomyelitis. The preserved dorsal skin of the middle finger provided local coverage of the palmar defect without the need for a separate donor site. The short documented follow-up does not permit conclusions regarding long-term hand function or sustained freedom from recurrence.

This case underscores the importance of considering fungal pathogens in progressive necrotizing wounds after environmental or occupational trauma. Early recognition, repeated microbiological sampling, appropriate systemic antifungal treatment, aggressive removal of nonviable tissue, and identification of previously unrecognized predisposing conditions may all contribute to successful management.

## 4. Conclusions

Post-traumatic *A. fumigatus* infection of the hand is rare but can result in extensive soft-tissue destruction and loss of digits. Fungal infection should be considered when a traumatic wound develops progressive necrosis or fails to respond as expected to conventional antibacterial treatment.

In the present case, repeated microbiological isolation of *A. fumigatus* established the clinical significance of the organism, while previously unrecognized uncontrolled diabetes mellitus was identified during hospitalization. Successful management required systemic antifungal therapy and repeated surgical source control. Three sequential debridements were followed by a fourth procedure combining further debridement, amputation of the irreversibly damaged middle and ring fingers, and immediate local flap reconstruction.

Early diagnosis and close cooperation between surgical, microbiological, and infectious disease teams are important in limiting tissue loss and achieving definitive infection control in these uncommon but potentially destructive infections.

## 5. Learning Points

1. Fungal infection should be considered in progressive necrotizing wounds following contaminated occupational or agricultural trauma, particularly when the clinical course is atypical or refractory to antibacterial therapy.
2. Repeated isolation of *A. fumigatus* from deep-tissue specimens in the setting of progressive tissue necrosis supports clinically significant infection rather than superficial colonization.
3. The absence of a previously known predisposing condition does not exclude host susceptibility; previously undiagnosed diabetes mellitus may become apparent during investigation.
4. Extensive post-traumatic Aspergillus infection may require repeated surgical debridement in addition to systemic antifungal therapy.
5. In cases of irreversible digital destruction, amputation and reconstructive coverage may be combined during definitive surgical management once adequate debridement has been achieved.

## Data Availability

The data supporting the findings of this study are included within the article. Further data are not publicly available due to patient privacy and confidentiality.
